# Supplementary material for: Are carbon emissions trading and green financial instruments synergistic? -Comprehensive quantitative research based on content analysis
Source: PLoS One. 2024 Mar 7;19(3):e0298601. doi: 10.1371/journal.pone.0298601 (PMC10919601; doi:10.1371/journal.pone.0298601)
Supplement: S1 Data — (ZIP) [file pone.0298601.s001.zip › Data/地级市Z_F工作报告.zip!/║■─╧/│╗╓▌╩╨/└┤╫╘╣1⁄2╓┌║┼í╛┬φ┐╦╩2╛▌═°í┐_2016.docx]

各位代表：

现在，我代表市人民政府，向大会报告工作，请予审议，并请市政协委员及其他列席人员提出意见。

一、2015年和“十二五”时期政府工作回顾

2015年，市人民政府在省委、省政府和市委的坚强领导下，紧密团结和依靠全市人民，主动适应经济发展新常态，积极应对经济下行压力持续增大等挑战，围绕稳增长、调结构、促改革、惠民生、防风险，大力实施“产业主导、全面发展”总战略，扎实推进四个“四年行动计划”，全力以赴“兴产业、强实体、提品质、增实效”，认真抓实市四届人大四次会议确定的各项任务，取得了积极成效。

(一)经济增长在精准施策中缓中趋稳。全年实现地区生产总值2012.1亿元，同比增长8.5%;规模工业增加值增长7.2%;一般公共预算收入220.3亿元，下降8.4%;城镇、农村居民人均可支配收入分别为25534元、11778元，分别增长8.1%、9.2%。全市固定资产投资2169亿元，增长19.5%，投资拉动经济增长4.4个百分点。郴州烟叶复烤厂异地技改、金贵白银城、中远泓广物流、黄莽公路等一批重大项目竣工，莽山水库、长鹿国际旅游休闲度假区、飞天山旅游文化产业园、人民东路延伸段、武广高铁郴州西站扩建、郴州机场大道等一批重大项目开工。全市社会消费品零售总额809.6亿元，增长12.2%。商品房销售面积、销售额分别增长20.6%、21%。消费拉动经济增长4.1个百分点。全市新增贷款165.3亿元，实现各类融资280.5亿元。政府债务风险防控有效，成功争取105.6亿元置换债券额度，市本级财政筹集政府偿债准备金11亿元。

(二)产业发展在转型升级中提质增效。全市高加工度工业、高技术工业增加值分别增长8.9%、6%。新增风力发电装机77.2万千瓦。工业技改投资523.3亿元。八达玻璃厂关闭并启动搬迁。万元规模工业增加值能耗下降16%。嘉禾县被授予“中国锻造之乡——绿色锻造基地”称号。强力推进创新创业园区“1555”工作计划，园区规模工业增加值占比达76%。郴州高新区升格为国家级高新区。桂阳有色金属产业园挂牌省有色金属重点产业园。国家有色贵重金属产品质检中心(湖南)通过验收。新增中国驰名商标5件、国家地理标志保护产品2件。临武通天玉湖南省地方标准实施。接待游客人数、旅游总收入分别增长15.4%、17.2%。北湖区列入全国休闲农业与乡村旅游示范县。东江湖景区成功创建国家5A级景区。第三届国际矿博会、第三届湘南投洽会和2015中国(湖南)红色旅游文化节、中国郴州(国际)休闲旅游文化节成功举办。湖南唯一的国家级国际性展会——国际矿博会永久性落户郴州。湘南国际物流园成功晋级首批省级示范物流园区。电子商务蓬勃发展，阿里巴巴等知名电商进驻郴州，郴州经开区获批省级电子商务示范基地。 “市场建设新三年行动计划”有序推进。资兴市、临武县农商行挂牌开业。汝城县金晋农牧在“新三板”挂牌上市。 “米袋子”“菜篮子”工程扎实有效。粮食总产量190万吨。收购烟叶100万担。农村土地流转规范有序。现代庄园经济加快发展。农副产品加工业增加值增长9.7%。成功举办首届湖南(郴州)特色农博会。汝城县、桂东县跻身全国重点产茶百强县。

(三)改革开放在重点突破中全面推进。认真贯彻落实中央、省委和市委改革部署，实施7大类77项改革，7项国家级、28项省级改革试点顺利推进。市、县(市、区)政府机构改革和乡镇区划调整全面完成，事业单位分类改革稳步推进。积极推进预算绩效管理改革、市直单位预决算信息全面公开，实行产业引导资金竞争性分配，资金使用效益进一步提高。投融资体制改革加快推进，莽山水库纳入国家第一批PPP模式试点水利项目。行政审批制度改革继续巩固深化，投资项目在线审批监管平台试运行，商事登记制度改革试点任务基本完成， “三证合一、一照一码”登记制度全面实施。国有林场改革任务基本完成。海关、口岸、检验检疫等功能性开放平台作用进一步增强，郴州铁路口岸内陆“无水港”建设积极推进。突出承接对接和战略合作，坚持精准招商，强化以商招商、产业链招商，成功引进深圳正威集团、华瀚科技控股、北京顺峰集团、重庆同元集团等一批战略投资者。全市实际利用外资13.5亿美元、到位内资408.2亿元，外贸进出口总额28.2亿美元。

(四)城乡面貌在统筹发展中显著变化。城市总体规划修改方案基本完成，专项规划体系更加健全，控制性详规覆盖面进一步拓宽。规划编制和执法加快向农村延伸。市中心城区实施基础设施项目171个，完成投资107.9亿元。郴州国际会展中心项目荣获中国建筑工程“鲁班奖”。依法持续开展城市管理和交通秩序整治，市容市貌持续巩固提升。“两供两治” 完成投资22.8亿元。东江湖引水一期工程进展顺利。成功创建湖南省节水型城市。各县城和示范镇建设加快推进。资兴市国家新型城镇化综合试点建设顺利推进。桂东县纳入省级新型城镇化试点县。宜章县、安仁县列入国家智慧城市试点县。桂东县沙田镇列入全国建制镇示范试点镇。汝城县大坪镇、嘉禾县龙潭镇被评为全国第四批文明村镇。县域经济加快发展。统筹城乡发展“十大示范工程”进展顺利。农村客运公交化改造加快推进。农村环境整治和规范农村村民建房成效显著。创建全国绿化模范城市通过验收。入选中国50大“氧吧”城市。安仁县获评全国生态魅力县。西河、永乐江国家湿地公园创建成功。嘉禾国家森林公园通过评审。狮子口省级自然保护区获批。全国水生态文明建设试点工作扎实有效。完成水利投资51.5亿元。解决41.9万农村人口的饮水不安全问题。中小河流治理、病险水库除险加固、农田水利建设进一步加强。140公里的西河生态休闲风光带全线建成。东江湖、青山垅—龙潭水库获批国家水利风景区。

(五)社会大局在共建共享中和谐稳定。“民生100工程”年度任务全面完成，全市财政民生支出占一般公共预算支出76.9%。全力推进精准扶贫，减少贫困人口7.23万人。新增城镇就业6.74万人、农村劳动力转移就业4.98万人。城乡居民养老、医疗保障水平进一步提高，在全省率先实现城乡居民医疗保险一体化管理。城乡低保水平和农村分散供养五保户供养标准进一步提高。新改扩建乡镇敬老院18所、县级福利院1所，新建社区居家养老服务中心40个、农村幸福院126个。“两房两棚”建设加快推进。市城区新增学位11420个。创建209所村小合格学校。普通高中发展水平提升。北湖区、桂东县创建为湖南省教育强县。郴州职业技术学院顺利通过省示范性(骨干)高职院校验收。创建体育之城加快推进，成功举办2015中国国际女排精英赛等多项国际赛事和市第一届运动会。我市被确定为国家体育产业联系点。公共文化服务体系进一步完善，农村广播“村村响”工程全面完成。“飞天·苏仙”大型山水实景演出成功推出。县级公立医院改革全面启动实施，基本药物制度实现全覆盖。公共卫生服务均等化步伐加快，医疗、卫生服务水平进一步提升。苏仙区被评为全国中医药先进单位。计生服务管理改革取得实效。食品药品安全保障有效。民族宗教、外事侨务、对台事务、统计、物价、人防、档案、气象、地震、水文、科普、地方志和妇女儿童、老龄人、残疾人、慈善等事业取得新进步。驻郴解放军和民兵预备役部队为地方发展作出新贡献。“六五”普法任务全面完成。公共法律服务体系建设试点稳步推进。安全生产态势平稳向好。公路“治超”成果持续巩固。“六打一治”专项行动深入开展，社会综治民调全省排名第一位。一批信访突出问题得到妥善解决。精神文明建设成效显著，涌现了全国道德模范刘真茂等一批先进模范人物。我市入选2015“中国最具幸福感城市”。

在推进经济社会发展、保障改善民生的同时，我们全面贯彻依法治国重大方略，大力简政放权，加快转变职能，严格按照法定权限和程序用权履职，建立并落实“三清单一目录”，行政效能和服务质量持续提升。坚持科学民主决策，强化决策落实，严格绩效考核，政府执行力进一步提升。坚持严格规范公正文明执法，强化对关系群众切身利益、群众反映强烈的重点领域的执法，维护了社会公平正义。强化公共资金预算管理、财政投资评审和审计监督，市本级“三公”经费下降9.45%，政府资金使用效益进一步提高。认真执行市人大及其常委会决议决定，自觉接受市人大、市政协的监督，注重发挥各民主党派、工商联、无党派人士的参政议政作用，支持工会、共青团、妇联等群团组织充分发挥桥梁纽带作用。扎实开展“三严三实”专题教育，狠抓廉政建设和作风转变，依法依纪查处了一批腐败案件，严肃了政风政纪，赢得了人民群众的普遍支持。

各位代表!2015年是“十二五”的收官之年。总结一年的工作，我们深感欣慰;回顾五年的发展，我们更加自豪。五年来，我们按照“四个走在全省前列”的要求，大力实施“一化两城三创四大”战略，取得了一系列重大发展成果，地区生产总值年均增长11.4%，全面小康实现程度由75.8%提高到89.6%(初步测算)，经济增速和全面小康推进工作综合排名均居全省前列，连年获得全省综合绩效考核先进表彰，是全市经济实力提升最快、城乡面貌变化最大、人民群众得实惠最多的时期之一。

——这五年，是我市综合实力大幅提升的五年。全市地区生产总值、工业增加值、一般公共预算收入分别从1000亿元、500亿元、100亿元台阶，迈上了2000亿元、1000亿元、200亿元台阶，培育了有色金属千亿产业、郴州高新区千亿园区;累计完成固定资产投资7368亿元，是“十一五”时期的3.4倍;累计新增专利授权4424件、中国驰名商标15件、国家地理标志保护产品4件，分别是“十一五”时期的2.3倍、7.5倍、4倍;桂阳籍女子射击运动员易思玲勇夺2012年伦敦奥运会第一块金牌。

——这五年，是我市城乡面貌发生巨变的五年。全市新建成高速公路474公里、国省干线公路900公里、农村公路3327公里，98%的建制村通客运班车;“大十字”城镇群基本形成，我市纳入全省新型城镇化试点市;成功创建国家园林城市、国家卫生城市、国家交通管理模范城市，荣获“全国文明城市提名城市”称号，成功承办一系列国际性体育赛事，城市知名度和美誉度大幅提升。

——这五年，是我市发展活力显著增强的五年。全市累计实际利用外资49.3亿美元、内联引资1576.5亿元、外贸进出口总额155.1亿美元，分别是“十一五”时期的2.5倍、2.2倍、5.4倍;成功引进一批包括世界500强企业在内的战略投资者;扎实推进了一批国家和省级改革试点，市场主体和创新创业活力显著增强。

——这五年，是我市生态文明建设成果丰硕的五年。全市森林覆盖率提高4.2个百分点，新增国家森林公园4个、国家湿地公园3个;成功创建国家森林城市，列入全国生态文明示范工程试点市、全国水生态文明城市建设试点市。在不少地方被视为“奢侈品”的青山绿水、蓝天白云、清新空气，已经成为郴州人民的“日用品”。

——这五年，是我市民生福祉极大改善的五年。全市财政民生支出由2010年的116.5亿元增加到291.3亿元，占一般公共预算支出的比重由72.3%提高到76.9%;城镇和农村居民人均可支配收入分别从15342元、5208元增加到25534元、11778元，累计31.52万人脱贫;县城以上城区累计新增义务教育学位10.9万个;安全生产形势平稳，社会大局和谐稳定，山川安澜、人民安居，公众对干部队伍的满意度稳居全省前列，社会综治民调连续五年居全省前两位。惠民政策的“红包”、改革发展的“红利”，不断“飞入寻常百姓家”。

五年砥砺前行，五年辉煌发展。五年来，我们既经历了产业转型、结构调整的“阵痛”，也收获了城乡巨变、民生提质的喜悦;既承受了经济下行、增速放缓的压力，也增强了科学发展、全面小康的信心。特别是，我们在产业转型升级、行政审批改革、生态文明建设、干部作风转变、城市提质、交通建设管理等很多方面，探索了“郴州模式”，创造了“郴州经验”，产生了“郴州效应”，创响了“郴州品牌”。开放的郴州，已经进入改革发展的“黄金时代”。510多万郴州人民，正加快向全面小康社会迈进!

成绩的取得，是省委、省政府和市委正确领导、科学决策的结果，是全市人民团结一致、奋力拼搏的结果，是市人大、市政协监督支持、积极参与的结果。在此，我代表市人民政府，向全市广大干部群众，向人大代表、政协委员，向各民主党派、工商联、无党派人士和人民团体，向驻郴部队官兵和中省驻郴单位，向离退休老同志，向所有关心支持郴州发展的各界朋友，表示衷心的感谢并致以崇高的敬意!

五年的探索实践，我们深刻地感到：办好郴州的事情，推进郴州的发展，建设美丽郴州，造福郴州人民，必须立足郴州实际，创新发展理念。深刻认识发展不足、发展不优的基本市情，坚持发展第一要务，牢固树立“产业主导、全面发展”理念，大力推进产业转型升级，着力提高发展的质量和效益，用发展的办法解决困难、化解矛盾;必须发挥郴州优势，抢占发展高地。充分发挥郴州作为湖南“南大门”和“一带一部”重要支点城市的区位优势，以及革命老区、扶贫片区、湘南承接产业转移示范区的政策优势，全面深化改革，扩大双向开放，强化创新驱动，敢于下好“先手棋”、发展新业态、追求高品质，努力把区位优势转化为开放优势、把政策优势转化为发展优势;必须突出郴州特色，打造发展亮点。牢牢把握郴州绿色生态这一突出特色，把生态文明理念贯穿经济社会发展全过程，在“五化同步”中牢牢守住生态底线，做好山水文章，彰显生态品牌，以城镇群为主要形态，统筹城乡发展，建设宜居宜业的现代山水名城;必须汇聚郴州力量，增强发展合力。始终牢记人民对美好生活的向往就是我们奋斗的目标，坚持以人为本，扎实保障和改善民生，持续深入开展作风建设，密切党群干群关系，让人民群众更多更公平地享受发展成果，在共建共享中凝聚人心、汇聚力量。这些行之有效的经验和做法，我们将继续坚持和发扬。

同时，我们也清醒地看到，政府工作还存在不少困难和问题。主要是：受宏观经济环境疲软不振、下行压力持续增大、发展基础仍不牢固等因素影响，地区生产总值等部分经济指标没有实现预期目标，稳增长的压力突出;经济发展仍未摆脱资源路径依赖，市场主体不足不优，自主创新能力不强，转型发展任重道远;城乡、区域发展还不平衡，居民持续增收困难，公共服务水平还不高，改善民生和脱贫攻坚任务艰巨;自然生态系统和环境基础脆弱，重点行业、区域、流域的环保形势仍然严峻;政府简政放权、职能转变还不到位，消极腐败现象仍然存在，干部办事效率和服务发展能力有待持续提升，廉政建设需要持续加强。对这些困难和问题，我们将高度重视，认真解决。我们坚信，有省委、省政府和市委的坚强领导，任何困难都压不倒开放包容、求实创新的郴州人民! 二、“十三五”时期政府工作基本思路和2016年工作安排

“十三五”时期，是我市产业转型升级的关键期、全面深化改革的攻坚期、全面建成小康社会的决胜期。这一时期，国家将加快实施“三大发展战略”，加快推进“五化同步”、贫困地区脱贫攻坚和革命老区开发建设。郴州作为湖南的开放前沿地和湘南起义革命老区、罗霄山扶贫片区，既是全省发展新格局中的重要“一极”，又是“一带一部”、“京广高铁经济带”的重要支点城市，一系列重大政策将产生叠加效应，我们仍处于可以大有作为的重要战略机遇期。

根据市委“十三五”规划建议，今后五年我市经济社会发展的指导思想是：高举中国特色社会主义伟大旗帜，全面贯彻党的十八大和十八届三中、四中、五中全会以及省委十届十五次全会精神，以中国特色社会主义理论为指导，深入贯彻习近平总书记系列重要讲话精神，按照“四个全面”战略布局，坚持发展第一要务，牢固树立创新、协调、开放、绿色、共享发展理念，以提高发展质量和效益为核心，加快形成引领经济发展新常态的体制机制和发展方式，大力实施“产业主导、全面发展”总战略，统筹推进经济建设、政治建设、文化建设、社会建设、生态文明建设和党的建设，促进新型工业化、新型城镇化、农业现代化、信息化和绿色化协调发展，努力把郴州打造成湖南深化改革开放的先行区、产业转型发展的示范区、统筹城乡和生态文明建设的引领区，确保全面建成小康社会，加快建设“实力、创新、开放、生态、人本”郴州。

按照上述指导思想，我们紧密结合郴州实际，编制了我市《“十三五”规划纲要》(草案)，提请会议审议。“十三五”时期，全市经济社会发展的主要预期目标是：地区生产总值年均增长9%左右;规模工业增加值年均增长9.5%左右;第三产业增加值年均增长12%以上;固定资产投资年均增长17%左右;社会消费品零售总额年均增长12%;外贸进出口总额年均增长15%;一般公共预算收入和地方财政收入年均均增长8%;城镇、农村居民人均可支配收入年均分别增长9%、10%;城镇登记失业率控制在4.6%以内;城镇化率58%;单位生产总值能耗累计下降15%，主要污染物排放总量明显下降;提前两年实现地区生产总值、城乡居民收入比2010年翻一番，提前一年全面建成小康社会。

在具体工作中，我们将把 “五个郴州”建设作为重中之重来抓。一是坚持产业主导、协调发展，建设“实力郴州”。把产业建设作为经济工作的重中之重，以四个“四年行动计划”为总抓手，加快转方式、调结构，推动传统优势产业改造提升，战略性新兴产业迅速发展，充分挖掘潜在增长能力，全力打造郴州产业升级版，不断提升郴州综合实力。二是坚持深化改革、创新发展，建设“创新郴州”。抓实全面深化改革和创新驱动发展战略，打破体制机制束缚，营造大众创业、万众创新的政策环境和制度环境，在全社会形成浓厚的创新氛围，凝聚强大的创新合力，让郴州成为生机勃发、活力迸发的创新创业之城。三是坚持内外融合、开放发展，建设“开放郴州”。主动融入国家“一带一路”、长江经济带发展战略，把全面对接珠三角摆在更加突出的位置，坚定不移地推进开放发展，全力打造便利通达、开放包容、合作共赢的基础设施条件和投资创业环境，加快构筑区域开放合作新高地。四是坚持两型引领、绿色发展，建设“生态郴州”。把生态文明建设放在更加突出的战略位置，坚持依法保护、源头治理、综合施策，着力推进绿色低碳发展，加快形成节约资源和保护环境的空间格局、产业结构和生产生活方式，全面构建生态文明新格局。五是坚持以人为本、共享发展，建设“人本郴州”。把脱贫攻坚作为“十三五”期间头等大事和第一民生工程，全力推进精准扶贫、精准脱贫，促进社会充分就业，完善社会保障体系，全面发展社会事业，推进基本公共服务均等化和社会管理创新，深入推进平安创建，不断增强人民群众的获得感和幸福感。

各位代表!九层之台，起于垒土;千里之行，始于足下。2016年是“十三五”的开局之年，我们要认真贯彻落实中央、省委和市委经济工作会议精神，围绕“十三五”总体发展目标，坚持稳中求进工作总基调，以抓铁有痕、踏石留印的作风，扎实做好各项工作，切实为“十三五”发展开好局、起好步。2016年全市经济社会发展的主要预期目标是：地区生产总值增长9%;规模工业增加值增长8%;第三产业增加值增长13%;固定资产投资增长18%;社会消费品零售总额增长12.5%;外贸进出口总额增长10%;实际利用外资增长10%;一般公共预算收入和地方财政收入均增长6%;城镇、农村居民人均可支配收入分别增长8%、9%;城镇登记失业率控制在4.6%以内;完成省里下达的节能减排目标。围绕上述目标，重点抓好七个方面的工作。

(一)突出兴产业、强实体，加快推动转型升级

围绕“兴产业、强实体、提品质、增实效”，大力实施“市场主体培育工程”，多举措帮助企业降低成本，加快提升产业整体素质和竞争力，不断提高供给体系的质量和效率。推进工业转型升级。充分发挥2个国家级质检中心作用，深化有色金属“五个一”战略体系建设，大力化解过剩产能，推进“两化”深度融合，加快有色、化工、建材、锻铸造、食品烟草等传统优势产业向高端化、智能化、绿色化发展。大力推进电子信息、先进装备制造、新材料、新能源等产业发展，重点培育壮大LED、矿物宝石、生物医药、大数据等新兴产业。深入实施创新创业园区“1555”工作计划，完善园区发展考核指标体系，促进园区经济增量提质。力争郴州出口加工区升级为综合保税区，郴州经开区和资兴经开区创建国家经开区取得突破。推进服务业高端化发展。加快以 “厕所革命”为突破口的旅游公共服务体系建设，继续推进“中国温泉之城”和“自驾自助游示范基地”建设。积极创建“休闲城市”。支持宜章县创建莽山国家级旅游度假区、汝城县创建热水温泉国家级旅游度假区。全面提升乡村旅游品质。推进与远大住工战略合作，抓好西河生态休闲风光带旅游开发。支持资兴市、苏仙区、桂东县创建国家级全域旅游示范区。承办好中国湖南首届国际美丽乡村生态休闲旅游节。以郴州国际会展中心为主体，整合各类场馆资源，完善会展组织服务体系，精心承办好第四届国际矿博会、第四届湘南投洽会、第二届湖南(郴州)特色农博会，推动会展经济更快发展。创新流通组织形式，培育支持流通市场主体和龙头企业发展。大力推行保障性住房、征地拆迁货币化安置，积极化解房地产库存，严格规范房地产市场管理，推进房地产业健康稳定发展。积极争创全国新农村住宅产业化示范城市。着力提高物业管理水平。完善金融组织体系，优化金融生态环境，提高金融服务实体经济、实体企业的效率。加强对中小微企业、农村特别是贫困地区的金融服务。大力推进保险业发展。加强社会诚信体系建设。大力发展体育产业、文化创意、网络服务、数字动漫、健康养老养生等新型服务业态。推进农业现代化。实施最严厉的耕地保护制度，切实加强耕地抛荒治理。抓好农村土地承包经营权确权登记颁证，规范农村土地流转，加快培育新型农业经营主体，促进农业适度规模经营。重点发展粮油、果茶、蔬菜、烤烟等“十大农业产业”，稳定粮食总产190万吨，收购烟叶105万担以上。大力推进郴州国家农业科技园区建设。抓好北湖石盖塘、资兴罗围、桂阳芙蓉等农产品加工园区建设，延长农业产业链条，提升农产品附加值。抓好郴州大道庄园经济、苏仙区现代休闲农业、资兴市花卉园林、桂东和汝城县茶叶等农业示范园区建设，推进农业规模化、集约化、标准化发展。落实粮食收储任务，健全供应保障体系，确保粮食安全。抓好农业科技服务、农产品质量安全和动植物疫病防治，提升现代农机服务水平。推进网络经济发展。推动实施大数据工程，在生产、消费、政务、民生等领域全面实施“互联网+”计划，支持基于互联网的各类创新，促进一、二、三产业融合发展。推进与知名电商战略合作，大力发展电子商务，加快郴州东谷电商产业园、郴州国际电商谷等电商基地建设，培育一批电子商务示范平台和企业，争取获批跨境电子商务试点。加快推进“宽带乡村”示范工程，让农村群众更好地领略世界的精彩、融入世界的发展。

(二)突出促投资、旺消费，着力扩大有效需求

坚持把促投资、旺消费作为重要支撑，大力培育新增长点，推动有质量、有效益、可持续发展。促进有效投资增长。紧盯国家、省“十三五”发展重大战略、重大工程、重大行动、重大计划，精心谋划，做实基础，力争更多项目进入国家、省项目计划“笼子”，最大程度地争取国家投资。搭建多形式的政银企对接平台，推动信贷资金更多地向实体经济倾斜、向项目建设集聚。继续实施“引老乡建家乡”工程，充分发挥商会的平台作用，积极参加中、省重大经贸活动，按照“五有”要求，大力开展专题招商、以商招商、产业链招商，重点引进世界500强、重点央企、知名民企，在更高起点上推进产业承接和企业联大靠强。强化签约项目跟踪落实，提高招商引资实效。创新投融资方式，认真落实扩大民间投资的准入政策，全面激活社会投资。引导和支持企业运用各类工具进入资本市场融资。组织县市区实施上市企业“破零”工程，促成一批企业上市或在“新三板”挂牌。强力推进项目建设。全市拟安排总投资1亿元以上重大建设项目1287个，年度计划投资1683亿元。重点推进金旺铋业深加工材料基地、东江湖大数据中心、长鹿国际旅游休闲度假区、飞天山旅游文化产业园等575个产业建设项目;武广高铁郴州西站扩容提质改造、人民东路延伸段、郴州机场大道、中石化新疆煤制天然气外输管道(新粤浙线)、市城区地下综合管廊(一期)等305个基础设施项目;市“四馆一厅”、郴州技师学院改扩建、郴州康复医院等334个民生和社会发展项目;东江湖生态环境保护与综合利用、鲁塘独立工矿区搬迁改造及转型发展等73个生态环保项目。全力争取郴州机场尽早开工。继续抓好兴永郴赣铁路、城市轻轨以及三南、黄长、临连、桂新高速公路等重大项目前期工作。加强依法征地拆迁和安置，强化重大项目用地保障，清理规范中介服务，持续优化项目建设环境。着力扩大消费需求。完善收入分配制度改革的配套措施，多渠道促进城乡居民增收，提高居民消费能力。加快实施“市场建设新三年行动计划”，推进农产品批发市场、商贸物流园区和标准化配送中心建设，强化市场秩序监管，切实改善消费环境。加强新一代信息基础设施建设，全面推进“三网融合”，积极扩大信息消费。加快升级旅游休闲消费，提升文体教育消费，促进健康养老养生和家政消费。加快“两房两棚”建设，落实好贷款降息、住房公积金政策调整等政策，促进住房消费。

(三)突出增动力、激活力，全面推进改革创新

坚持以深化改革促进扩大开放，以扩大开放促进深化改革，大力实施创新驱动发展战略，为经济社会发展注入新动力、增添新活力。抓实全面深化改革的各项任务。深化行政审批制度改革，提高政府效能。进一步明晰市与区事权责任，理顺市与区发展机制，更好地调动市、区两级推动发展的积极性。深化国资管理体制改革，促进政企政资分开，完善现代企业制度。深化要素配置市场化改革，促进人才、资金、技术等有序流动。深化公共资源管理体制改革，加快建立统一、开放、竞争、有序的公共资源市场交易体系。深化开放型经济改革，加快建立开放型经济新体制。深化财税体制改革，健全全口径预算管理体系，推进政府性债务预算编制，稳步推进“营改增”、消费税、资源税等税制改革。加快推进安仁、永兴、桂阳、嘉禾等县农村信用联社改制。深化以农村集体产权制度、农业经营制度为核心的农村综合改革。深化供销合作社综合改革。完善户口登记政策，推进以满足新市民需求、购租并举为主要方向的住房制度改革，积极探索农业转移人口市民化，加快提高户籍人口城镇化率。全面启动司法行政体制改革。稳步推进不动产登记改革，确保年底前初步建立统一登记制度。积极稳妥推进自然资源生态空间统一确权登记工作。全面完成市级党政机关公务用车改革。推进更宽领域更高水平的双向开放。以建设“无水港”城市为核心，优化口岸服务，提升口岸功能，力争郴州公路口岸跻身国家一类口岸行列。加快推进口岸物流中心、国际快件监管中心扩容、往来港澳公路跨境快速通关等项目建设，加快融入粤港澳通关一体化。支持国际快件个人物品清关业务发展，做大做强国际快件业务，确保“五定班列”正常运行。加快“两平台一基地”建设。高位推动湘粤开放合作试验区建设取得实质性突破。推动外贸转型升级，促进服务贸易等外贸新业态发展。引导支持企业到境外发展。大力推进大众创业万众创新。启动实施“关键技术突破工程”。加快建设永兴国家稀贵金属再生利用高新技术产业化基地，推进稀贵金属深加工技术突破。支持建设国家钨深加工技术研发中心、开发钨化工产品及钨合金材料。支持“微晶石墨提纯技术”成果应用及产业化，加快石墨烯产品研发和技术攻关。加快建设环保产业成果转化基地，转化一批重金属污染防治、矿山生态修复和资源综合回收利用等关键技术。积极创建国家知识产权示范城市。大力推进创业带动就业。深化商事登记和“三证合一、一照一码”制度改革，支持风险投资、创业投资和创业担保贷款发展，引导社会资金和金融资本支持创业活动。支持农民工返乡创业，发展新型农业经营主体。加强创新创业指导服务。统筹推进高校毕业生等重点群体就业。引导农村劳动力有序外出和就地就近转移就业。加快发展市场化、专业化、集成化、网络化的众创空间，为创业者提供低成本、便利化、全要素、开放式的综合服务平台和发展空间。推进郴州高新区、郴州经开区科技企业孵化器试点示范园区建设，力争郴州经开区创建为国家级创业孵化基地。

(四)突出人为本、一体化，扎实抓好城乡统筹

尊重城镇发展规律，以人的城镇化为核心，着力提升城镇发展水平，统筹推进城乡一体化发展。大力发展县域经济。把因地制宜壮大特色产业作为统筹城乡发展的重要支撑，支持永兴特色制造业省重点县、临武特色农副产品加工产业省重点县和资兴、汝城、宜章文化旅游产业省重点县建设。完善城乡规划体系。强化规划引领，推进城市总体规划修改，启动空港新城总体发展规划编制，加快编制中心城区道路交通整治规划、“大十字”城镇群轨道交通线网规划，推进中心城区综合管廊和海绵城市建设规划编制，完善市场布局、养老设施、体育设施等其它专项规划，积极推进“多规合一”。完善县城、村镇规划。严格规划执法，确保违法建设“零增长”。提高城镇建管水平。市中心城区拟安排基础设施建设项目166个、年度计划投资84.7亿元。着力完善公共服务和生活配套，加快城市新区繁荣发展。突出抓好交通互联互通，促进新老城区融合发展。扎实推进“棚户区改造三年行动计划”，加快下湄桥、南塔、东河、文星路等片区棚户区改造。积极推进城乡垃圾分类及收转运体系一体化建设。大力推进“两供两治”，加快东江湖引水一期工程和“气化郴州”步伐，启动实施东江湖引水二期工程(桂阳段)。着力抓好交通拥堵问题综合治理。加快创建“公交都市”。强化“门前三包”落实，更好地发挥“数字城管”的作用，推进城市精细化管理。持续推进“大十字”城镇群“五同工程”建设，加速郴资桂、郴永宜融合发展。抓好县城和示范镇、特色镇建设。支持嘉禾县全省城乡一体化体制机制创新示范区建设。加快建设美丽乡村。持续推进农村公路建设和养护管理提质，加快农村客运班线公交化改造。加快莽山、桃源、茶安、三百蹬、黄口堰、来洋洞等水库建设。全面完成全国水生态文明建设三年试点工作任务。大力推进农村安全饮水巩固提升、农田水利建设、中小河流治理，突出抓好防汛抗旱和重要水源地保护。继续抓好农网改造升级。支持发展农村集体经济。注重生态环境保护、历史文化传承和体现乡土特色，探索农村环境综合整治长效机制，深入推进规范农村村民建房，统筹抓好农村危房改造、“空心村”整治，加强古村落保护，确保在新农村建设中“多留遗产、少留遗憾”。

(五)突出提品质、守底线，抓牢抓实生态保障

全面推进“新三创”，牢牢守住生态底线，推动形成绿色发展方式和生活方式，用绿水青山带来金山银山。继续推进城乡绿化建设。大力实施 “绿城攻坚”活动(六期)、城市绿荫行动(二期)和“市城区立体绿化三年行动计划”。全面推进以交通干线、西河沿线和市城区周边可视山头为重点的生态景观提质。切实抓好植树造林、矿山复绿和石漠化、“天坑”、裸露山地治理，持之以恒抓好“五禁”措施的落实。扎实推进环境综合治理。全面实施湘江污染防治第二个“三年行动计划”，加强三十六湾、玛瑙山、柿竹园及周边等重点地区重金属污染综合治理，加快东江湖保护和翠江、舂陵江流域综合整治项目实施。实行最严格水资源管理制度。全力推进大气污染防治行动计划，进一步提升区域大气环境质量。切实抓好节能减排降耗。实施环境质量和污染排放总量双控，加强污染物排放精细化减排管理，全面实施排污权有偿使用和交易制度。突出抓好重点行业、重点领域的节能减排。引导和鼓励绿色低碳出行。大力发展循环经济与清洁生产，集约节约利用资源能源。支持资兴国家循环经济示范市、永兴国家循环经济示范园、桂阳国家循环化改造示范试点园区发展。大力构筑生态安全屏障。严格落实主体功能区制度，严守资源消耗上限、环境质量底线、生态保护红线。扎实推进国家级南岭山地森林及生物多样性重点生态功能区示范试点、东江湖流域生态红线制度改革试点建设，加快构建“一湖两山三水”生态屏障。加强自然保护区和珍稀动植物保护。通过推进绿色发展，使郴州处处呈现出人与自然和谐相处的美丽画卷。

(六)突出见实效、可巩固，全力开展脱贫攻坚

用好片区扶贫和革命老区建设政策，广泛调动各方面力量，扎实推进“五个一批”工程和“十大帮扶”措施，更加注重对特定人群特殊困难的精准帮扶，确保10万人口脱贫。发展生产脱贫一批。抓好产业园、念好“山”字经、唱好旅游戏、用好互联网、打好生态牌，因地制宜发展特色产业。着力抓好茶叶、油茶、花卉苗木、中药材等种植基地建设、龙头企业培育和农产品流通，促进特色农业发展。着力引进一批劳动力吸纳能力强、符合生态环保要求的劳动密集型企业，让更多的贫困农民就近就地就业。争创全国乡村旅游脱贫示范区。切实抓好以小额信贷扶贫为重点的金融扶贫，确保全年发放扶贫小额信贷5亿元以上。加强贫困地区基础设施建设，改善生产生活条件。全力争取6月底前厦蓉高速赤石特大桥通车。易地搬迁脱贫一批。对很难实现就地脱贫的贫困人口，分年度、有计划地组织实施易地搬迁，制定并落实后续扶持措施，确保搬得出、稳得住、能致富。实施“农村危房改造精准扶贫四年行动计划”。继续做好永兴县、汝城县移民避险解困搬迁全国试点工作。生态补偿脱贫一批。加大贫困地区生态保护修复力度，积极争取重点生态功能区转移支付，扩大政策实施范围，让有劳动能力的贫困人口就地转成护林员等生态保护人员。发展教育脱贫一批。财政教育经费继续向贫困地区、基础教育、职业教育倾斜，帮助贫困地区改善办学条件。对建档立卡的家庭经济困难普通高中学生，全部免除学杂费。对农村家庭幼儿特别是留守儿童给予特殊关爱。全面实施“一户一产业工人”培养计划，加强农民职业技能和实用技术培训。社会保障兜底一批。对完全或部分丧失劳动能力的贫困人口，实行社会保障兜底，统筹农村扶贫和低保标准，加大其它形式的社会救助力度。加强医疗保险和医疗救助，新型农村合作医疗和大病保险政策向贫困人口倾斜。引导和支持有劳动能力的贫困人口不等不靠、自力更生、勤劳致富。只要我们咬定目标，苦干实干，就一定能够打赢脱贫攻坚战，让全市所有贫困人口一道迈入全面小康社会!

(七)突出惠民生、促和谐，不断增进人民福祉

始终把人民的利益放在首位，把群众的冷暖放在心上，继续实施“民生100工程”，着力在社会保障、文化教育、医疗卫生、公共安全、交通出行、养老服务、强农惠农等方面，解决一批与群众日常生活息息相关的问题，让人民群众得到实实在在的好处。推进社会保障扩面提质。全面推进全民参保登记工作。积极开展园区养老保险缴费费率过渡试点。探索建立城乡统一的居民基本医疗保险制度。有效推进城乡居民省内异地就医联网即时结算。加强社会救助、城乡低保、慈善和减灾救灾等工作。继续实施农房保险和农业保险。健全新型养老服务体系，引导民办养老机构发展。加强保障性住房筹集和管理，让住房困难群众早日实现安居梦想。全面发展社会事业。进一步完善城乡义务教育经费保障机制，加快村级合格学校创建，实施“市中心城区中小学化解大班额三年行动计划”，突出抓好乡村教师队伍建设，推进义务教育均衡发展。逐步普及高中阶段教育，促进普通高中优质发展。加强职业教育基础能力建设，优化职业院校布局和专业结构，分类推进中等职业教育免除学杂费。全面实施第二期“学前教育三年行动计划”。推进特殊教育发展。支持在郴高等院校和郴州技师学院发展。鼓励引导民办学校发展。强化学校安全管理。加强公共文体设施、服务体系建设。鼓励创作文艺精品。大力发展群众文化体育事业。积极创建国家历史文化名城和体育之城。加强医疗服务与公共卫生管理体系建设。巩固完善国家基本药物制度。实施“市中心城区医疗卫生服务发展三年行动计划”。抓好重点传染病预防控制。加快中医药事业发展。鼓励、引导、规范社会办医。落实一对夫妇可生育两孩政策，提高生殖健康、妇幼保健、托幼管理等公共服务水平，促进人口均衡发展。加强城乡社区建设。抓好建制村合并和第十次村委会换届工作。组织开展好第三次全国农业普查工作。认真做好广播影视、民族宗教、外事侨务、对台事务、物价、统计、文物、档案、气象、地震、水文、科普、地方志、人防和妇女儿童、老龄人、残疾人、慈善等工作。充分发挥工会、共青团、妇联等群团组织的桥梁纽带作用。深入推进军民融合发展，大力加强全民国防教育，落实军人军属优抚政策，密切军政军民关系。营造和谐社会环境。严格落实 “一岗双责”，持续推进安全生产提升年“十大战役”，深化“打非治违”和隐患排查治理，确保安全生产形势持续稳定向好。完善监管体制机制，严格食品药品安全监管。推进社会治安网格化管理，加快智能“天网”工程建设，严厉打击各类违法犯罪，争创全国综治工作先进市。依法规范互联网管理，净化网络环境。加强信访基层基础工作，进一步规范信访秩序。深化以人民调解为基础的“大调解”工作，积极化解影响社会稳定的突出矛盾。持续巩固公路治超成果。强化公共应急管理。完善社会求助体系，加强政府法律援助，维护社会公平正义。

各位代表!面对新形势、新任务、新目标和人民群众的新期盼，我们必须忠实践行“三严三实”要求，振奋精神、奋发有为，强化责任担当，加快建设服务政府、责任政府、法治政府、廉洁政府。把“三严三实”贯穿于政府工作始终。坚持“严”字当头、“实”字托底，以“三严”祛歪风，以“三实”聚正气，用“三严三实”的标准全面考量政府工作，切实解决“不严不实”的突出问题。坚持简政放权、放管结合、优化服务，加快转变政府职能。完善在线审批监管平台，畅通行政审批服务“绿色通道”。简化公共服务流程，方便群众办事。完善综合绩效考核，加强督查督办，确保政令畅通。对于忽视民生、为官不为的“庸政”“懒政”，坚决从严整治、追责到位，不断提升政府执行力。把依法行政贯穿于政府工作始终。坚持科学民主决策，严格按照法定权限和程序行使权力、履行职责，全面落实“三清单一目录”制度。坚持严格规范公正文明执法，严格行政执法责任和过错追究制度。严格执行市人大及其常委会的决议决定，自觉接受市人大及其常委会、市政协的监督，不断提高人大代表建议和政协提案办理质量，主动听取民主党派、工商联、无党派人士和人民团体的意见。把廉政建设贯穿于政府工作始终。全面落实党风廉政建设“两个责任”，认真贯彻执行《中国共产党廉洁自律准则》和《中国共产党纪律处分条例》，依纪依法惩治腐败，驰而不息纠正“四风”，严肃查处群众身边的腐败问题和损害群众利益的不正之风。强化政府内部层级监督，进一步加强行政监察和审计监督，严格对权力集中部门和岗位的监督。深入推进政务公开和财政预决算、“三公经费”和审计结果公开，加强廉政风险防控，有效预防腐败行为发生，以党风廉政建设和反腐败斗争的实效取信于民。

各位代表!新的蓝图已经绘就，进军号角已经吹响。让我们紧密团结在以习近平同志为总书记的党中央周围，在中共郴州市委的正确领导下，团结和依靠全市人民，抢抓机遇，坚定信心，顽强拼搏，为全面建成小康社会、加快建设“五个郴州”而努力奋斗!
